# The impact of prescription drug insurance on cost related non-adherence to medications in Canada: A Heckman sample selection approach

**DOI:** 10.1371/journal.pone.0289776

**Published:** 2023-08-09

**Authors:** Qi Zhang, Audrey Laporte

**Affiliations:** 1 Institute of Health Policy, Management and Evaluation, University of Toronto, Toronto, ON, Canada; 2 Canadian Center for Health Economics, Toronto, ON, Canada; Universidad Del Rosario, COLOMBIA

## Abstract

Unlike some other high-income counties, Canada does not provide universal prescription drug coverage. The various extent of coverage may left some Canadians vulnerable to cost-related non-adherence (CRNA) to medications. Using data from the 2015 national cycle of the Canadian Community Health Survey, we examine the impact of having private and public drug coverage on mitigating the risk of CRNA with a logit model and a Heckman selection model. CRNA was only observed in respondents who had prescriptions to fill, and respondents did not randomly make decisions on whether to get a prescription. This results in a classic sample selection problem. We found a higher estimated probability of reporting CRNA for uninsured respondents from the Heckman selection model than from the logit model. Respondents with government coverage only had a slightly higher probability of reporting CRNA relative to respondents with private coverage. These findings suggest that, without accounting for sample selection, the risk of not having drug insurance coverage is likely to be underestimated. Moreover, despite covering a less healthy cohort of respondents, the government insurance plans reduce risk of CRNA to a comparable level with private insurance.

## Introduction

Prescription drug plays a crucial role in treating and preventing disease, particularly for people with chronic conditions [1]. Meanwhile, the costs associated with prescription drug are rapidly increasing in Canada. For example, Statistics Canada states that Canada’s drug price are now the third highest among Organization for Economic Co-operation and Development (OECD) countries [2]. Additionally, the Canadian Institute for Health Information (CIHI) reports that prescription drug costs are growing faster than hospital and physician costs [3].

However, unlike the necessary hospital and physician costs, which are covered by a universal health insurance plan, prescription drug costs are not [4, 5]. Expenses for prescription drugs are financed through a mixed structure consisting of public and private drug insurance plans and out-of-pocket payments [6]. In 2018, public drug spending accounted for 43.4% total spending of the prescription drug expenditure, with the private insurance and out-of-pocket payment accounted for 36.8% and 19.8% respectively [7].

For instance, Canada’s three largest provinces, namely British Columbia, Ontario, and Quebec, differ significantly in terms of the public drug coverage they offer to their residents [8]. In British Columbia, the coverage plan (Fair PharmaCare) is based on income, meaning that all families are eligible, but those with lower net incomes receive more coverage than those with higher ones. This coverage plan includes all age groups and offers high deductible coverage to all households except for those with low incomes. In Ontario, the main coverage plan (Ontario Drug Benefit, ODB) is age-based and provides low deductible coverage to residents aged 65 and older, and cost-free coverage to children and youth under 25 who are not covered by a private plan. In Quebec, the coverage plan is private-public, with premium-based public coverage (with monthly deductible, coinsurance, and monthly and yearly maximum contributions) for those without private insurance. Moreover, the employment-based private plans has been mandatory for eligible employees [9].

No Canadian province other than Quebec mandates private drug insurance for employees who qualify for extended health benefits as part of their compensation packages. However, many Canadians in all other provinces have private insurance on a voluntary basis as part of the compensations packages. In private insurance plans, deductibles are infrequent and only apply to plans covering 11% of Canadians who have private insurance. However, the majority of Canadians with private drug insurance pay co-insurance (67% of all beneficiaries) or fixed co-payments (17% of beneficiaries).

Recent studies show that there is significant disparities in private prescription drug coverage caused by household income gradient [10, 11]. Individuals of higher socioeconomic status are more likely to have private drug coverage, wile those of lower socioeconomic status are more likely to have public coverage or no coverage [12]. The standard for publicly funded prescription drug coverage is set at the provincial level; and the public programs usually cover elders, people in the government welfare programs, or people with high prescription drug costs compared to their household income [13]. As a result, there is a wide spectrum of generosity levels in term of prescription drug coverage, based on individuals’ socioeconomic status and province of residence [14].

Due to varying degrees of coverage among individuals in Canada, prescription drug costs can be a heavy financial burden for certain patients. There is evidence suggest that patients take economic considerations into account when making decisions about their medication, which is referred to as cost-related non-adherence (CRNA) [15]. Extensive research in the health services literature has examined the issue of patients being forced to skip their medications, because they do not have adequate prescription drug insurance or they do not have insurance at all and they cannot afford out-of-pocket payments [16–23]. In addition to the lack of insurance coverage, a wide variety of socioeconomic factors have been associated with an increased risk of reporting CRNA including low income, young age, chronic illness, province of residence, immigrant status, not having a regular primary physician, being a woman, being a sexual minority and having less education [14, 24–29].

Previous research in the Canadian context mostly lumps private insurance and government insurance together as having prescription drug coverage. This approach cannot provide evidence to evaluate the government funded programs against the private insurance plans. Moreover, earlier studies either included chronic conditions as a numeric category or were conducted on a group of patients with a specific condition, such as obesity. It is reasonable to hypothesize that not all chronic conditions have the same impact on CRNA. Therefore, certain chronic conditions are expected to cause higher risk to CRNA, but others are not. Most importantly, there is an overlooked sample selection problem associated with the single equation model (e.g. logit or probit) to study CRNA within people who have at lest one prescription. The economic literature generally reports that insurance coverage has a significant effect on increasing health services utilization, and the use of services is responsive to the scheme of the insurance plan [30–32]. These findings suggest that people do not make decisions about whether to get prescriptions at random, and factors such as insurance status affect the probability of getting a prescription. Therefore, not adjusting the probability difference of getting a prescription associated with different individuals leads to inconsistent and biased estimates.

The main objective of this study is to examine the impact of holding private and public drug coverage on mitigating CRNA by taking into account of the sample selection problem. More specifically, CRNA is only observed in individuals who have obtained a prescription, and the probability of receiving a prescription is influenced by factors such as insurance status and other socioeconomic variables. It is reasonable to hypothesize that the sample selection problem of getting a prescription does exist, and not correcting it is likely to lead to underestimated risks of reporting CRNA for individuals without coverage. This paper also extends the current literature by including a more comprehensive set of controls for chronic conditions, as well as some previously neglected variables that are important in determining CRNA such as household size and mental health status. Firstly, the impacts of prescription drug insurance status on CRNA are examined using a conventional logit model to serve as baseline results. Secondly, a two stage probit model with sample selection originated from Heckman selection model is exploited to adjust for selection bias [33]. The results provide evidence that corroborates the hypotheses. In addition, having either public or private coverage significantly reduced the risks of reporting CRNA. We turn now to the study’s methods.

## Data and methods

To address the research question, data was extracted from the Public Use Microdata File of 2015 Canadian Community Health Survey (CCHS) produced by Statistics Canada, a cross-sectional survey that that is representative of the national population aged 12 years or more living in private dwellings [34]. The survey includes extensive self reported information on demographic, social, and economic correlates of health as well as measures of health status. Research Ethics Board approval is not required for analyses using publicly available datasets.

### Analytical sample

**Fig 1:**
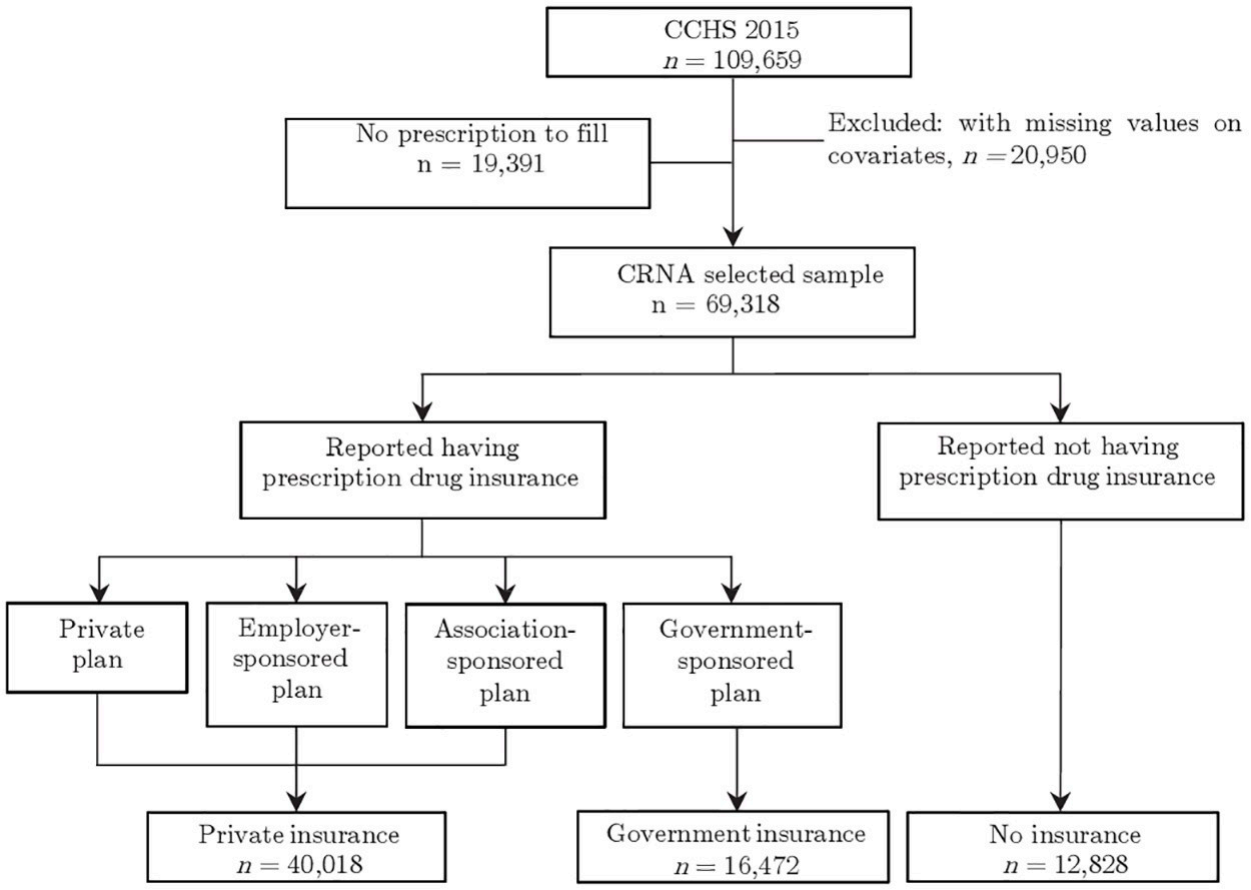
Selection of analytical sample from the CCHS 2015.

Of the 109,659 respondents in the 2015 CCHS, 88,709 had valid and non-missing responses to all study variables. As Fig 1 demonstrates, 69,318 out of 88,709 respondents received at least one prescription in the past 12 month. The report of CRNA, the outcome variable, is only observed within this selected sample, and previous studies did not address this selection problem. One of the key explanatory variables of interest, the insurance status is also shown in Fig 1. Within the sample of which CRNA can be observed, 57.7% (40,018 out of 69,318) respondents self reported as having private insurance that covered at least part of prescription drug cost. There were a small number of respondents were covered by both private and government plans. Without enough detailed information to further distinguish these individuals, they were classified as holding private plans. The proportion of respondents covered solely by the government sponsored medication plans and uninsured were 23.8% and 18.5% respectively. In summary, the analytical sample contains 88,709 respondents for a Heckman selection model, and a selected portion of 69,318 respondents to be fitted with a logit model in line with previous studies for comparison. The details are more fully described in the following sections.

Table 1 provides evidence that the probability of getting a prescription varies with different insurance status. Only 72.83% of the uncovered respondents received a prescription which is significantly lower than the covered counterparts. The proportion of respondents with private insurance and government insurance who got at least one prescription is at 77.15% and 85.67%, respectively. This result suggests that there may be a sample selection bias. Insurance status not only affects one’s probability of reporting CRNA but also the probability of receiving a prescription. Without a prescription, the CRNA is never observed. In other words, only respondents with at least one prescription will be selected in the sample to study CRNA, and this sample selection process may not happen randomly. As a result, estimating coefficients only on the basis of respondents with prescriptions from a single equation model, like logit, leads to biased and inconsistent estimates.

**Table 1:** Probability of having at least one prescription by insurance status.

Sample with no missing value (88,709)				
Had prescription	No insurance	Private	Government	p-value
No	27.17%	22.85%	14.33%	<0.001
	(4,786)	(11,850)	(2,755)	
Yes	72.83%	77.15%	85.67%	
	(12,828)	(40,018)	(16,472)	
Total	100%	100%	100%	
	(17,614)	(51,868)	(19,227)	

p-value from Chi-square test to examine if there is a relationship between insurance status and whether had prescription to fill.

Among the 69,318 respondents who received prescriptions, Table 2 shows the probability of reporting CRNA. The percentage of respondents who reported CRNA and have no drug insurance is significantly higher than those with private or government insurance. The numbers are 13.53% for uncovered individuals compared to a lower 3.92% and 6.49% for the covered counterparts. A Chi-square test was conducted for the data presented in Tables 1 and 2, and the observed differences in both tables are significant.

**Table 2:** Probability of having at least one prescription by insurance status.

Sample with a prescription to fill (69,318)				
CRNA	No insurance	Private	Government	p-value
No	86.47%	96.08%	93.51%	<0.001
	(11,093)	(38,415)	(15,403)	
Yes	13.53%	3.92%	6.49%	
	(1,735)	(1,567)	(1,069)	
Total	100%	100%	100%	
	(12,828)	(40,018)	(16,472)	

p-value from Chi-square test to examine if there is a relationship between insurance status and CRNA.

### Study variables

Our measures of cost-related non-adherence (CRNA) use responses to one question in the survey. The respondent is asked whether didn’t fill or skipped a prescription due to cost in the past 12-month. The responses could be one of the three options: (1) Yes, (2) No, and (3) No prescription to fill in the last 12-month. These three responses are equivalent to asking two sequential questions. First, respondents were asked if they had a prescription to fill in the last 12 months. Then, those who had received a prescription were asked the question whether they did not fill a prescription or skipped does of medication because of cost.

The independent variable of primary interest is insurance status, classified into three categories: private insurance, government insurance and no insurance. The included control variables are in accordance with the conception frameworks on healthcare access, and were employed by previous research studied the same topic using earlier versions of CCHS [24, 35]. These include province of residence, sex, age, education attainment, household income, and self-reported physical health status. Some other control variables empirically tested as important to CRNA were constructed using the survey: household size, self-reported mental health status, whether having a regular healthcare provider, immigration status, dwelling status, chronic conditions and whether a language other than English and French is the primary language at home [36]. It is worth noting that chronic conditions were included as an array of binary variables, given the relative large sample size. The specific chronic conditions included are asthma, sleep apnea, arthritis/ scoliosis/ fibromyalgia, back problem, high blood pressure, high blood cholesterol, heart disease, stroke, diabetes, cancer, and mood/anxiety disorder. Description of independent and dependent variables by whether received prescription can be found in Table 3.

**Table 3:** Description of dependent and independent variables, by prescription.

Variable	Without prescription	With prescription	Total	p-value
	N = 19,391	N = 69,318	N = 88,709	
CRNA	NA	4,371 (6.3%)	NA	NA
*Drug insurance status*				<0.001
No insurance	4,786 (24.68%)	12,828 (18.51%)	17,614 (19.86%)	
Private(ref.)	11,850 (61.11%)	40,018 (57.73%)	51,868 (58.47%)	
Government	2,755 (14.21%)	16,472 (23.76%)	19,227 (21.67%)	
*Has post-secondary education*				<0.001
Yes (No as ref.)	12,270 (63.28%)	41,921 (60.48%)	54,191 (61.09%)	
*Province*				<0.001
NL	406 (2.09%)	2,227 (3.21%)	2,633 (2.97%)	
PE	237 (1.22%)	1,221 (1.76%)	1,458 (1.64%)	
NS	730 (3.76%)	3,190 (4.60%)	3,920 (4.42%)	
NB	446 (2.30%)	2,197 (3.17%)	2,643 (2.98%)	
QC	3,764 (19.41%)	15,278 (22.04%)	19,042 (21.47%)	
ON(ref.)	5,868 (30.26%)	20,380 (29.40%)	26,248 (29.59%)	
MB	1,056 (5.45%)	3,382 (4.88%)	4,438 (5.00%)	
SK	828 (4.27%)	2,982 (4.30%)	3,810 (4.29%)	
AB	2,653 (13.68%)	8,362 (12.06%)	11,015 (12.42%)	
BC	2,574 (13.27%)	8,843 (12.76%)	11,417 (12.87%)	
YT	185 (0.95%)	514 (0.74%)	699 (0.79%)	
NT	253 (1.30%)	470 (0.68%)	723 (0.82%)	
NU	391 (2.02%)	272 (0.39%)	663 (0.75%)	
*Sex*				<0.001
Male(ref.)	10,828 (55.84%)	29,278 (42.24%)	40,106 (45.21%)	
Female	8,563 (44.16%)	40,040 (57.76%)	48,603 (54.79%)	
*Age*				<0.001
18-24	2,233 (11.52%)	4,253 (6.14%)	6,486 (7.31%)	
25-39	6,094 (31.43%)	13,850 (19.88%)	19,944 (22.48%)	
40-54	5,415 (27.93%)	15,395 (22.21%)	20,810 (23.46%)	
55-64	3,105 (16.01%)	13,924 (20.09%)	17,029 (19.20%)	
65+(ref.)	2,544 (13.12%)	21,896 (31.56%)	24,440 (27.55%)	
*Household size*				<0.001
Hsize1(ref.)	4,910 (25.32%)	21,407 (30.88%)	26,317 (29.67%)	
Hsize2	6,159 (31.76%)	27,327 (39.42%)	33,486 (37.75%)	
Hsize3	3,097 (15.97%)	8,713 (12.57%)	11,810 (13.31%)	
Hsize4	3,279 (16.91%)	7,839 (11.31%)	11,118 (12.53%)	
Hsize5+	1,946 (10.04%)	4,032 (5.82%)	5,978 (6.74%)	
*Household income*				<0.001
$0-20,000	1,584 (8.17%)	7,041 (10.16%)	8,625 (9.72%)	
$20,000-39,999	2,934 (15.13%)	13,325 (19.22%)	16,259 (18.33%)	
$40,000-59,999	3,152 (16.25%)	11,681 (16.85%)	14,833 (16.72%)	
$60,000-79,999	2,726 (14.06%)	9,563 (13.80%)	12,289 (13.85%)	
$80,000+(ref.)	8,995 (46.39%)	27,708 (39.97%)	36,703 (41.37%)	
*Perceived mental health*				<0.001
1.Poor	101 (0.52%)	1,118 (1.61%)	1,219 (1.37%)	
2.Fair	688 (3.55%)	4,195 (6.05%)	4,883 (5.50%)	
3.Good	3,772 (19.45%)	16,661 (24.04%)	20,433 (23.03%)	
4.Very good	7,340 (37.85%)	25,495 (36.78%)	32,835 (37.01%)	
5.Excellent(ref.)	7,490 (38.63%)	21,849 (31.527%)	29,339 (33.07%)	
*Perceived health*				<0.001
1.Poor	146 (0.75%)	2,542 (3.67%)	2,688 (3.03%)	
2.Fair	786 (4.05%)	7,555 (10.90%)	8,341 (9.40%)	
3.Good	4,181 (21.56%)	21,120 (30.47%)	25,301 (28.52%)	
4.Very good	7,742 (39.93%)	25,156 (36.29%)	32,898 (37.09%)	
5.Excellent(ref.)	6,536 (33.71%)	12,945 (18.67%)	19,481 (21.96%)	
*Has a regular healthcare provider*				<0.001
Yes (No as ref.)	13,646 (70.37%)	61,132 (88.19%)	74,778 (84.30%)	
*Was born in Canada*				<0.001
Yes (No as ref.)	15,689 (80.91%)	58,829 (84.87%)	74,518 (84.00%)	
*Neither English nor French as primary language at home*				<0.001
Yes (No as ref.)	1,365 (7.04%)	2,836 (4.09%)	4,201 (4.74%)	
*Owns home*				<0.001
Yes (No as ref.)	13,879 (71.57%)	50,709 (73.15%)	64,588 (72.81%)	
*Has asthma*				<0.001
Yes (No as ref.)	800 (4.13%)	6,741 (9.72%)	7,541 (8.50%)	
*Has sleep apnea*				<0.001
Yes (No as ref.)	523 (2.70%)	4,923 (7.10%)	5,446 (6.14%)	
*Has arthritis/scoliosis/fibromyalgia*				<0.001
Yes (No as ref.)	2,752 (14.19%)	23,440 (33.82%)	26,192 (29.53%)	
*Has back problem*				<0.001
Yes (No as ref.)	2,616 (13.49%)	16,663 (24.04%)	19,279 (21.73%)	
*Has high blood pressure*				<0.001
Yes (No as ref.)	1,003 (5.17%)	18,825 (27.16%)	19,828 (22.35%)	
*Has high blood cholesterol*				<0.001
Yes (No as ref.)	855 (4.41%)	12,859 (18.55%)	13,714 (15.46%)	
*Has high heart disease*				<0.001
Yes (No as ref.)	266 (1.38%)	5,200 (7.5%)	5,468 (6.16%)	
*Has diabetes*				<0.001
Yes (No as ref.)	276 (1.42%)	7,411 (10.69%)	7,687 (8.67%)	
*Suffers from a stroke*				<0.001
Yes (No as ref.)	55 (0.28%)	1,095 (1.58%)	1,150 (1.30%)	
*Diagnosed with cancer*				<0.001
Yes (No as ref.)	732 (3.77%)	6,389 (9.22%)	7,121 (8.03%)	
*Has mood/anxiety disorder*				<0.001
Yes (No as ref.)	1,084 (5.59%)	10,974 (15.83%)	12,058 (13.59%)	

The reference level is indicated as ref. p-value from Chi-square test to examine the independence of whether had a prescription to fill and other control variables.

Table 3 demonstrates that whether a respondent had a prescription to fill is unlikely happened randomly.In addition to differences in insurance status, there are significant differences in nearly all aspects, particularly in perceived health status and whether the respondent has certain chronic conditions. A corrective method for sample selection need be applied to these respondents received prescription from whom the dependent variable can be observed.

### Statistical analysis

To estimate the impact of insurance status on reducing the risk of experiencing CRNA, a logit model is estimated using the sample of respondents who received a prescription. The weighting of respondents are adjusted using weights provided in 2015 CCHS to reflect the overall representativeness in the population [2]. Then, a bivariate probit model with sample selection is employed to mitigate the selection bias associated with not fully observed CRNA. To compare results from logit and bivariate probit regression models, the average partial effects (APE) are reported, along with the odds ratios. The APE is obtained by averaging all of the in-sample estimated partial effects [37]. The bivariate probit model model contains an outcome equation and a selection equation [38]. The outcome equation is shown in Eq (1):

$$\begin{array}{c} y_{j}=x_{j}\beta +u_{1j} \end{array}$$ (1)

$$\begin{array}{c} \begin{array}{c} y_{j}=\{\begin{array}{c} \text{1,}\;\text{if}\;\text{reports}\;\text{experienced}\;\text{CRNA}\;\text{in}\;\text{the}\;\text{past}\;\text{year}\\ \text{0,}\;\text{if}\;\text{reports}\;\text{did}\;\text{not}\;\text{experience}\;\text{CRNA}\;\text{in}\;\text{the}\;\text{past}\;\text{year} \end{array} \end{array} \end{array}$$

where **x** ***β*** = *β*_1_*x*_1_ + *β*_2_*x*_2_ + ⋯*β*_*k*_*x*_*k*_, containing all independent variable described in Table 3.

However, the dependent variable, report of CRNA, was not always observed. Rather, CRNA for respondent j was observed only if the respondent received a prescription. The selection equation for respondents had at least one prescription in the past year satisfies the following condition

$$\begin{array}{c} y_{j}^{select}=z_{j}\gamma +u_{2j}>0. \end{array}$$ (2)

The error terms are assumed to have the following characteristics [38]:

$$\begin{array}{c} \begin{array}{c} u_{1}∼N\left(0,1\right),\\ u_{2}∼N\left(0,1\right),\\ \text{corr}\;(u_{1},u_{2})=\rho . \end{array} \end{array}$$

When *ρ* ≠ 0, estimates from applying standard logit/probit to the outcome equation are biased and inconsistent. However, simultaneously estimate the outcome equation and the selection equation using maximum-likelihood method yield consistent estimates. The log likelihood being maximized is:

lnL=∑j∈Pyj=1wjln{Φ2(xjβ+offsetjβ,zjγ+offsetjγ,ρ)}+∑j∈Pyj=0wjln{Φ2(-xjβ+offsetjβ,zjγ+offsetjγ,-ρ)}+∑j∉Pwjln{1-Φ(zjγ+offsetjγ)}

where *P* is the set of respondents who had prescription (i.e., *y*_*j*_ is observed),*w*_*j*_ is the sample weight for respondent j, Φ_2_(⋅) is the cumulative distribution function for bivariate normal, Φ(⋅) is the standard normal cumulative distribution function.

In order for this model to be well identified, the selection equation Eq (2) should not have the same set of independent variables as the outcome equation Eq (1) [33]. Put simply, there should be at least one variable in **z** that is not in **x**. From a theoretical standpoint, most of the explanatory variables in the outcome equation are actually identical to those in the selection equation. The unique explanatory variables to the selection function are whether a respondent consulted a general practitioner (family doctor) and/or a specialist in the past year. Consultations with physicians are likely to affect the probability of getting a prescription, and these consultations may not directly affect the probability of experiencing CRNA [39]. Therefore, two additional binary variables can be included in the selection equation that are not in the outcome equation, and their descriptions based on whether a prescription was received are shown in Table 4. All regression analyses were conducted using Stata/SE 15.1 with CCHS public use micro-data files and bootstrap weights provided by Statistics Canada.

**Table 4:** Description of independent variables only in selection equation, by prescription.

Variable	Without prescription	With prescription	Total	p-value
	N = 19,391	N = 69,318	N = 88,709	
*Consulted with general practitioner-past 12 months*				
Yes	8,986 (46.34%)	54,467 (78.58%)	63,453 (71.53%)	<0.001
No(ref.)	10,405 (53.66%)	14,851 (21.42%)	25,256 (28.47%)	
*Consulted with specialist-past 12 months*				
Yes	2,957 (15.25%)	24,950 (35.99%)	27,907 (31.46%)	<0.001
No(ref.)	16,434 (84.75%)	44,368 (64.01%)	60,802 (68.54%)	

p-value from Chi-square test to examine if there is a relationship between consult a physician and had a prescription to fill

## Results

The estimated correlation between the error terms of the outcome equation and the selection equation $\hat{\rho }$ is around -0.47 (-0.58 to -0.34 as in the 95% C.I.). Additionally, Table 5 presents the average partial effects (APE) based on estimates of the coefficients ***β*** from the logit model without correction and the Heckman selection model, along with the odds ratio [37].

**Table 5:** Estimated logit odds ratio and APE for both logit model and probit model with sample selection.

Variable	Logit without correction		Corrected probit
	Odds Ratio	APE	APE
*Drug insurance status: Private as ref*.			
No insurance	3.157 (0.000)	0.0783 (0.000)	0.1050 (0.000)
Government	1.281 (0.004)	0.0116 (0.006)	0.0145 (0.008)
*Sex: Male as ref*.			
Female	1.315 (0.000)	0.0153 (0.000)	0.0095 (0.030)
*Province: ON as ref*.			
NL	0.698 (0.008)	-0.0185 (0.004)	-0.0330 (0.000)
PE	0.841 (0.294)	-0.0095 (0.269)	-0.0201 (0.064)
NS	0.751 (0.021)	-0.0151 (0.014)	-0.0237 (0.003)
NB	1.029 (0.831)	0.0017 (0.833)	-0.0028 (0.782)
QC	0.781 (0.003)	-0.0133 (0.003)	-0.0207 (0.000)
MB	0.915 (0.434)	-0.0051 (0.425)	-0.0064 (0.432)
SK	0.894 (0.427)	-0.0063 (0.413)	-0.0087 (0.372)
AB	1.101 (0.258)	0.0059 (0.262)	-0.0050 (0.443)
BC	1.029 (0.730)	0.0017 (0.731)	-0.0013 (0.837)
YT	0.994 (0.978)	-0.0004 (0.978)	-0.0055 (0.738)
NT	0.528 (0.020)	-0.0296 (0.003)	-0.0385 (0.007)
NU	0.189 (0.000)	-0.0542 (0.000)	-0.0548 (0.001)
*Age: 65+ as ref*.			
18-24	4.018 (0.000)	0.0694 (0.000)	0.0967 (0.000)
25-39	3.876 (0.000)	0.0665 (0.000)	0.0900 (0.000)
40-54	2.670 (0.000)	0.0409 (0.000)	0.0586 (0.000)
55-64	2.065 (0.000)	0.0270 (0.000)	0.0391 (0.000)
*Household size: Hsize1 as ref*.			
Hsize2	1.207 (0.006)	0.0095 (0.005)	0.0130 (0.003)
Hsize3	1.410 (0.000)	0.0184 (0.000)	0.0248 (0.000)
Hsize4	1.504 (0.000)	0.0223 (0.000)	0.0305 (0.000)
Hsize5+	1.459 (0.002)	0.0204 (0.004)	0.0282 (0.001)
*Household income: $80,000+ as ref*.			
$0-20,000	2.260 (0.000)	0.0455 (0.000)	0.0643 (0.000)
$20,000-39,999	2.461 (0.000)	0.0521 (0.000)	0.0682 (0.000)
$40,000-59,999	1.997 (0.000)	0.0367 (0.000)	0.0504 (0.000)
$60,000-79,999	1.617 (0.000)	0.0233 (0.000)	0.0304 (0.000)
*Perceived health: Excellent as ref*.			
1.Poor	2.012 (0.000)	0.0455 (0.000)	0.0491(0.001)
2.Fair	1.605 (0.000)	0.0282 (0.000)	0.0217 (0.017)
3.Good	1.372 (0.002)	0.0178 (0.001)	0.0106 (0.126)
4.Very good	0.914 (0.348)	-0.0043 (0.356)	-0.0130 (0.034)
*Perceived mental health: Excellent as ref*.			
1.Poor	1.873 (0.001)	0.0390 (0.004)	0.0494 (0.005)
2.Fair	1.580 (0.000)	0.0267 (0.000)	0.0325 (0.001)
3.Good	1.252 (0.011)	0.0120 (0.011)	0.0132 (0.027)
4.Very good	1.215 (0.014)	0.0103 (0.013)	0.0113 (0.027)
*Has post-secondary education: No as ref*.			
Yes	1.084 (0.199)	0.0045 (0.199)	0.003 (0.465)
*Has a regular healthcare provider: No as ref*.			
Yes	1.000 (0.998)	0.0001 (0.998)	-0.0141 (0.043)
*Was born in Canada: No as ref*.			
Yes	0.871 (0.108)	-0.0078 (0.110)	-0.0139 (0.026)
*Neither English nor French as primary language at home: No as ref*.			
Yes	0.716 (0.023)	-0.0187 (0.023)	-0.0264 (0.014)
*Owns home:No as ref*.			
Yes	0.712 (0.000)	-0.0190 (0.000)	-0.0234 (0.000)
*Has asthma: No as ref*.			
Yes	1.373 (0.000)	0.0177 (0.000)	0.0163 (0.007)
*Has sleep apnea: No as ref*.			
Yes	1.102 (0.350)	0.0054 (0.350)	0.0034 (0.652)
*Has arthritis/scoliosis/fibromyalgia: No as ref*.			
Yes	1.292 (0.000)	0.0144 (0.000)	0.0142 (0.005)
*Has back problem: No as ref*.			
Yes	1.372 (0.000)	0.0177(0.000)	0.0202 (0.000)
*Has high blood pressure: No as ref*.			
Yes	1.027 (0.735)	0.0015 (0.735)	-0.0132 (0.037)
*Has high blood cholesterol: No as ref*.			
Yes	1.140 (0.135)	0.0074 (0.135)	0.0023 (0.713)
*Has high heart disease: No as ref*.			
Yes	0.764 (0.018)	-0.0151 (0.018)	-0.0245 (0.002)
*Has diabetes: No as ref*.			
Yes	1.266 (0.040)	0.0132 (0.041)	0.0061 (0.438)
*Suffers from a stroke: No as ref*.			
Yes	1.280 (0.334)	0.0139 (0.335)	0.0117 (0.537)
*Diagnosed with cancer: No as ref*.			
Yes	0.858 (0.133)	-0.0086 (0.133)	-0.0138 (0.050)
*Has mood/anxiety disorder: No as ref*.			
Yes	1.479 (0.000)	0.0219 (0.000)	0.0192 (0.001)
Log pseudolikelihood	-3.9e+6		-1.57e+7
Pseudo *R*^2^	0.138		

N = 69,318 and p-values in parenthesis were obtained with heteroskedastic robust standard errors.

Without drug insurance coverage, it seems to be the most important factor affecting one’s risk of experiencing CRNA. The APE estimate from the logit model indicates a staggering 7.83% higher probability of reporting CRNA, compared to people with private insurance plans. However, this figure is likely to be underestimated without correction for sample selection. Lack of insurance coverage affects people’s decision to seek a prescription at the first place. It is reasonable to assume that that the probability of receiving a prescription would be lower if out-of-pocket medication payments are unaffordable. After correcting for sample selection, the results show that the uncovered respondent were associated with a 10.5% higher probability of reporting CRNA compared to those covered by private plans. In contrast, respondents under government coverage were associated with slightly higher level of risk of reporting CRNA, with an increased probability of 1.16% and 1.45% from logit and Hekman selection model respectively, compared to respondents with private coverage.

The findings also indicate that female respondents and younger respondents were more likely to report CRNA. Several social-economic factors are associated with a higher risk of reporting CRNA including from a larger household, having a lower income household, and not owning a home.Lower self-perceived health/mental health was also linked to a higher risk of reporting CRNA. The relationship between CRNA and chronic conditions is a mixed picture, and is likely to be disease specific. For example, a respondent with a mood disorder or back problem was more likely to report CRNA, while another respondent with a heart problem was less likely to report having experienced CRNA. In the logit model, there were no significant relationship between CRNA and variables such as having a regular health care provider and was born in Canada. However, these variables are associated with lower risk of reporting CRNA in the corrected probit model.

## Discussion

Overall, our results from the logit model are consistent with an earlier study that found a 4.5 times higher odds of reporting CRNA among respondents without insurance coverage, compared to those with coverage [24]. After controlling for factors like household size and a variety of chronic conditions, we found that the odds of reporting CRNA among respondents who had no coverage was 3.16 times higher than those with coverage through private insurance. It is impossible to completely control for individual heterogeneity. Thus, one limitation of this study is that the estimates are not immune to omitted variable bias, and future studies could further explore this topic with panel data.

The needs for prescription of medication is not evenly distributed across the population. Respondents who had prescriptions tended to be older, have worse health and mental health status, have a regular health care provider, insurance coverage, and one or more chronic conditions. Therefore, among the population who do need prescribed medications, insurance status and chronic conditions appear to influence their decisions to visit a general practitioner or a specialist to obtain a prescription. Among the older and sicker population who have prescriptions, insurance status is likely to impact their decisions to fill their prescriptions. Thus, a Heckman selection model was appropriate to address this issue and could move us one step forward to obtaining consistent estimates. Another limitation should be acknowledged is that some assumptions of the Heckman sample selection model are over-simplistic and may not hold for the analytical sample. For example, the two error terms from the outcome and selection equation are unlikely to be normal.

Although respondents with government provided coverage were associated with a 1.45% higher probability of reporting CRNA from the corrected probit model, this result needs to be interpreted with caution. For people with government-provided coverage, sample selection to receive prescription might not work in the same direction as people with no coverage. Findings in Table 1 indicate that people with government insurance are more likely to obtain a prescription than uncovered people and people covered by private insurance. This may imply that, on average, people covered by public policies have greater medication needs than the other two groups. Neither the non-exhaustive list of chronic conditions nor the perceived health/mental health status is able to fully capture the health inequities among the three insurance status groups. Furthermore, due to data limitation, we cannot control the overall generosity level of the government insurance versus private insurance or the generosity level specific to each chronic condition in the study sample. In addition, the poor quality of some control variables and potential omitted variable bias could play an role in complicating the process to get consistent estimation. For example, the household income variable is categorical and censored above $80,000 which can not fully capture the income inequalities between people covered by private and government insurances. Considering these limitations on controlling income inequities, the government insurance may not be inferior to private insurance in terms of mitigating risk of experiencing CRNA. Taken together it is hard to determine if the 1.16% elevated probability of report CRNA from the logit model is biased upward or downward.

## Conclusion

This article aims to examine the impact of prescription drug insurance status on cost related no-adherence of medication in a large-scale survey, including a comprehensive set of health, social and demographic characteristics as controls. It is worth noting that this paper does not attempt to formulate a causal relationship between insurance status and the experience of cost-related non-adherence to medication. Instead, it provides new correlational insights by accounting for the selection bias arising from the different probabilities of getting a prescription associated with different insurance statuses. Comparing the estimation results between the logit model and the Heckman selection model suggests that the probability difference of getting a prescription among individuals should not be overlooked.

The main conclusion is that the risk of experiencing CRNA are likely to be underestimated for individuals with no insurance coverage, without adjusting for sample selection. Moreover, no insurance coverage may be the factor that has the most salient impact on CRNA based on our APE results, and it may also be the most transformable through public policy. The current government provided insurances significantly reduces the risk of CRNA compared to no coverage at all. More research is needed to investigate the trade off between increased costs associated with expanding the prescription drug coverage and the benefits of reducing expenditures in areas such as hospital admissions [40].

Some previously neglected factors were found to have significant association with CRNA including: household size, perceived mental health status, having a regular health provider, and speak a language other than English or French at home. Moreover, the findings also suggest that the risk of experiencing CRNA varies considerably across different chronic condition groups. However, more detailed information such as average cost per prescription, frequency of dosage and efficacy to disease are required to understand the underlining mechanisms of how would each specific chronic condition influences CRNA.
